# Transesophageal Echocardiogram with Contrast in Pulmonary Intravascular Dilation in Hepatosplenic Schistosomiasis

**DOI:** 10.5935/abc.20190234

**Published:** 2019-11

**Authors:** Sandra Nívea dos Reis Saraiva Falcão

**Affiliations:** 1 Universidade de Fortaleza (Unifor), Fortaleza, CE - Brazil; 2 Universidade Federal do Ceará, Fortaleza, CE - Brazil; 3 Hospital de Messejana Dr. Carlos Alberto Studart Gomes, Fortaleza, CE - Brazil

**Keywords:** Echocardiography, transesophageal/methods, Schistosomiasis, Hepatopulmonary Syndrome, Liver Injury, Chronic/physiopathology

The Hepatopulmonary syndrome (HPS) is defined as a defect in arterial oxygenation induced by intrapulmonary vascular dilation (IPVD) associated with liver disease. The vascular component includes diffuse or localized capillary dilation and, less commonly, pulmonary arteriovenous malformations.^1^

A sensitive approach for the early detection of altered arterial oxygenation is the calculation of the alveolar-arterial oxygen pressure difference (PA-aO_2_). This difference can be elevated before arterial oxygen pressure becomes abnormal.^2^ At sea level, PA-aO_2_ ≥ 15 mmHg is considered abnormal (it changes to 20 mmHg in individuals over 64 years).^3^

However, the increase in PA-aO_2_ alone is not sufficient for the diagnosis of HPS, as the presence of IPVD should also be present and it is defined when the pulmonary capillary diameter is 15 to 60 microns.^1^

The diagnosis of IPVD can be made using Technetium-99m (99mTc) labeled to human albumin aggregate particles or by pulmonary angiography. However, microbubble contrast-enhanced transthoracic echocardiography (cTTE) is a sensitive, noninvasive method, considered a gold standard.^4^

The cTTE uses microbubbles obtained from agitated saline solution and infused into a peripheral vein. Under normal conditions only the right heart chambers are filled by the contrast and the microbubbles are filtered into the pulmonary capillary bed, of which average diameter is approximately 10 micrometers. In the presence of IPVD, capillary dilation promotes shunting and allows the passage of the microbubbles to the left cardiac chambers approximately 4 to 6 beats after their appearance in the right chambers, and when this happens we consider the cTTE positive for the presence of IPVD.^5^ The presence of contrast in the left chambers may occur early (before 4 beats), in which case the presence of intracardiac shunt (intra-atrial defect or patent foramen ovale) is diagnosed.

The presence of microbubbles in left cardiac chambers can be graded as follows: grade 0, no bubbles present in the left atrium; grade I, few punctiform bubbles present in the left atrium; grade II, moderate bubbles without completely filling the left atrium; grade III, moderate bubbles completely filling the left atrium, but with less intensity than the right atrium; and grade IV, homogeneous distribution of bubbles in both atria.^5^

The expected grading in normal individuals, regarding microbubble contrast in the left chambers, is grade 0; however, in some patients the presence of contrast grade I is described, a fact explained, at least partially, by the presence of any physiological pulmonary shunts.^6^

The cTTE may be positive in up to 47% of patients with liver disease (with or without HPS), but only about 32-59% of these patients have arterial hypoxemia, which may be a preclinical or silent form of HPS.^7^

Vedrinne et al.,^5^ demonstrated the superiority of the transesophageal echocardiography with contrast (cTEE) in detecting the passage of bubbles to the left chambers and, consequently, IPVD, when compared with cTTE in patients with severe liver disease with liver transplant indication, being also safe in these patients, even in the presence of esophageal varices.^5^ Despite the advantage for visualization of microbubbles, cTEE requires sedation, is more expensive and carries a potential risk in patients with esophageal varices (which are frequent in patients with liver disease).^1^

Although HPS is often associated with cirrhosis, there is no correlation between the primary cause of liver damage and HPS,^8^ and the occurrence of the syndrome has been documented in patients with portal hypertension without cirrhosis.^9^ In schistosomiasis, a disease caused by *Schistosoma mansoni* infection and endemic in northeastern Brazil, portal hypertension may be present in 2-7% of cases,^10^ secondary to periportal fibrosis.

The prevalence of HPS in patients with liver cirrhosis on the transplantation list in Brazil is approximately 5 to 16%;^11^ however, there are few studies evaluating the presence of HPS in patients with schistosomiasis. In a study published by Ferreira et al.,^12^ the authors observed a 6% prevalence of HPS in patients with schistosomiasis.

In an article published in this issue about the use of cTEE in the diagnosis of IPVD,^13^ the authors demonstrated that cTEE showed an increase in the diagnosis of IPVD for patients with hepatosplenic schistosomiasis, similar to that observed in the literature for patients with liver cirrhosis. The present study is a pioneer in evaluating the presence of IPVD in this group of patients using cTEE.

A major challenge in performing a transesophageal examination in these patients is the presence of esophageal varices due to the potential risk of bleeding. It should be noted that in the study mentioned in this issue,^13^ all patients underwent digestive endoscopy, and, in the presence of esophageal varices, they were treated with sclerotherapy before the transesophageal examination. Despite the safety of the cTEE described by the authors, care should be taken to evaluate the presence or absence of esophageal varices and the need for sclerosis therapy to minimize the risk of bleeding.

The increase in the diagnosis of IPVD through cTEE brings up a group of patients with pulmonary vascular alterations diagnosed by echocardiography; however, with preserved arterial oxygenation profile, which may reflect a preclinical phase of the disease. This aspect, observed in the patients of this study with hepatosplenic schistosomiasis (HSS), has also been described in other studies with cirrhotic and non-cirrhotic patients.^1^ The prognostic role of this finding and its consequence in the natural course of the disease is unknown, requiring further observations.

Given the importance of schistosomiasis in our country and the lack of publications regarding the diagnostic analysis of HPS in patients with HSS, studies in this field should be appreciated for their pioneering characteristics and for contributing to a better understanding of this pathology.
